# BMC Surgery reviewer acknowledgement 2013

**DOI:** 10.1186/1471-2482-14-2

**Published:** 2014-01-16

**Authors:** Thomas A Rowles

**Affiliations:** 1 BioMed Central, Floor 6, 236 Gray’s Inn Road, London WC1X 8HB, UK

## Abstract

**Contributing reviewers:**

The editors of BMC Surgery would like to thank all our reviewers who have contributed their time to the journal in Volume 13 (2013).

## 

Javier A.-Cienfuegos

Spain

Saleh Abbas

New Zealand

Adib Abla

USA

Thad Abrams

USA

Ishag Adam

Sudan

Magnus Ågren

Denmark

Sarfraz Ahmad

United Kingdom

Annette B. Ahrberg

Germany

Donato Francesco Altomare

Italy

Emad Aly

United Kingdom

Ali Aminian

Iran

Evzen Amler

Czech Republic

Basil Ammori

United Kingdom

Roland Andersson

Sweden

Martin Angele

Germany

Luca Ansaloni

Italy

Amedeo Anselmi

Italy

Stavros Antoniou

Germany

Stanley Anyanwu

Nigeria

Theerachai Apivatthakakul

Thailand

Helmut Arbogast

Germany

Jorg Bahm

Germany

Gian Luca Baiocchi

Italy

Christina Bali

Greece

Zhihui Ban

USA

Virinder Bansal

India

Michal Barak

Israel

Mustafa Baskaya

USA

Hasan Batirel

Turkey

Dirk Bausch

Germany

Stefan Benz

Germany

Paul Bevis

United Kingdom

Aneel Bhangu

United Kingdom

Daniel Birch

Canada

Robin Blackstone

USA

Raoul Borioni

Italy

Federico Bottaro

Argentina

Nathan Boyer

USA

Michael Brauckhoff

Norway

Brandon Broome

USA

Steven Brown

United Kingdom

Walter Brunner

Switzerland

Martin Brutsche

Switzerland

Clifford Buckley

USA

Jakob Burcharth

Denmark

Pietro Giorgio Calo'

Italy

Stefania Camagni

Italy

Enzo Capocasale

Italy

Fausto Catena

Italy

Andres Cervantes

Spain

Phillipo Leo Chalya

Tanzania

Grigoris Chatzimavroudis

Greece

Steven Chen

USA

Ta-Liang Chen

Taiwan

Zheng Chen

USA

Piero Chirletti

Italy

Bruno Chrcanovic

Sweden

Ricardo Cohen

Brazil

Laura Collins

USA

Giuliana Cortese

Italy

Michele Cote

USA

Emma Culver

United Kingdom

Brian Davidson

United Kingdom

Mechteld De Jong

Netherlands

Marcioi De Moraes

Brazil

Emil Dediol

Croatia

Adel Denewer

Egypt

Federico Di Rocco

France

Salomone Di Saverio

Italy

Marius Distler

Germany

Ilgaz Dogusoy

Turkey

Peter Donkor

Ghana

Cornelia Dotzenrath

Germany

Dipesh Duttaroy

India

Sebastian Ekenze

Nigeria

Eric Elster

USA

Itaru Endo

Japan

Giorgio Ercolani

Italy

Eloy Espin

Spain

Ciro Esposito

Italy

Johannes Fakler

Germany

Walid Faraj

Lebanon

Stefan Farkas

Germany

Mazda Farshad

Switzerland

Angela M.R. Ferrante

Italy

Enrico Fiori

Italy

Jason B Fleming

USA

Michael Flierl

USA

Daniel Ford

USA

Randall Franz

USA

Shin Fujita

Japan

Sean Galvin

Australia

Amador Garcia Ruiz De Gordejuela

Spain

Michael Gardner

USA

Jens Peter Garne

Denmark

Jeff Garner

United Kingdom

Clemens Gerstenkorn

Germany

Sonja Gillen

Germany

Michela Giulii Capponi

Italy

David Glick

USA

Dhanny Gomez

United Kingdom

Matthias Goos

Germany

Peter Goretzki

Germany

Vincent Gremeaux

France

Bradley Gross

USA

Jodok Matthias Grueneberger

Germany

Ali Guner

Turkey

Thilo Hackert

Germany

David Hak

USA

Jason Hall

USA

Wulf Hamelmann

Germany

Jonathan Hardman

United Kingdom

Seetharaman Hariharan

Trinidad and Tobago

James Harrop

USA

Werner Hartwig

Germany

Alan Hemming

USA

Benoit Herbert

USA

Andrew Hill

New Zealand

Justus Hilpert

Germany

Jens Hoeppner

Germany

Zhiyong Hou

China

Chih-Kun Huang

Taiwan

Karel We Hulsewe

Netherlands

Abdulzahra Hussain

United Kingdom

Maurizio Iacobone

Italy

Hiroo Imazu

Japan

Leif Israelsson

Sweden

Hrvoje Ivekovic

Croatia

Beata Jabłońska

Poland

Sandeep Jain

India

Gigot Jean-François

Belgium

Zhiwei Jiang

China

Magnus Kaffarnik

Germany

Morton Kahlenberg

USA

Adrien Kaladji

France

Meletios Kanakis

Greece

Paul Karanicolas

Canada

Martin Karpeh

USA

Airazat Kazaryan

Norway

Tobias Keck

Germany

Dezsö Kelemen

Hungary

Rory Kennelly

Ireland

Ji Wan Kim

South Korea

Sungjoo Kim

South Korea

Heidi King

USA

Yoji Kishi

Japan

Olivia Kituuka

Uganda

Daniela Kniepeiss

Austria

Bülent Koçer

Turkey

Arto Kokkola

Finland

Gregory Kouraklis

Greece

Avdyl Krasniqi

Serbia

Simon Kuesters

Germany

Rekha Kumar

India

Prakash Kurumboor

India

Oren Lapid

Netherlands

Anna Lee

Hong Kong

Han Hong Lee

South Korea

Jandee Lee

South Korea

Christian Letoublon

France

Dileep Lobo

United Kingdom

Feizhou Lu

China

David Lumenta

Austria

Renato Lupinacci

France

Craig Lynch

Australia

Giovanni Maconi

Italy

Manuel Maglione

Austria

Philipp Manegold

Germany

Francesco Saverio Mari

Italy

Athanasios Marinis

Greece

Goran Marjanovic

Germany

Stephen Mark

New Zealand

Cyril Mauffrey

USA

Anthony Maxwell

United Kingdom

Edward Mcgillicuddy

USA

Kandace Mcguire

USA

Penelope Mcmanus

United Kingdom

Spencer Melby

USA

Abdul Sattar Memon

Pakistan

Anastasia Mentessidou

Greece

Michelangelo Miccini

Italy

Marco Milone

Italy

Tomica Milosavljevic

Serbia

Aytan Miranda Sipahi

Brazil

Rohin Mittal

India

Susan Moffatt-Bruce

USA

Prashant Mohite

United Kingdom

Michele Molinari

Canada

Umberto Morelli

France

Martin Mortazavi

USA

Michael Moser

Canada

Beat Peter Müller-Stich

Germany

Nicolas Murith

Switzerland

Myura Nagendran

United Kingdom

Youssef Narjis

Morocco

Atsushi Nashimoto

Japan

Farideh Nejat

Iran

Calvin Ng

Hong Kong

Haruo Nishijima

Japan

Nuria Novoa

Spain

Furuzan Numan

Turkey

Evarist Tm Nyawawa

Tanzania

Patrick O Dwyer

United Kingdom

Karl Oldhafer

Germany

Hisashi Onodera

Japan

Carlos Ordonez

Colombia

Daniel Orringer

USA

Akihiko Oshita

Japan

Georg Osterhoff

Switzerland

Jean O'Toole

USA

Moreica Pabbruwe

Australia

Javier Padillo

Spain

Giovanni Pallabazzer

Italy

Jaideep Pandit

United Kingdom

Jose Maria Pascual

Spain

Maciej Patrzyk

Germany

Douglas Paull

USA

Timothy Pawlik

USA

Evangelos Perdikakis

Greece

Frank Pfeffer

Norway

Elia Poiasina

Italy

Christophe Pomel

France

Marco Raffaelli

Italy

Ashwani Rajput

USA

Julie Reeve

New Zealand

Kerstin Reimers

Germany

Andreas W Reske

Germany

Cornelia Richters

Netherlands

Mårten Risling

Sweden

Andres Rodriguez-Lorenzo

Sweden

Jelle Ruurda

Netherlands

Shomei Ryozawa

Japan

Antoni Sabate

Spain

Attilio Carlo Salgarelli

Italy

Ricardo Santos De Oliveira

Brazil

Motoko Sasaki

Japan

Thomas Scalea

USA

Andreas Schnitzbauer

Germany

Beat Schnüriger

Switzerland

Martin Schuster

Germany

Andreas Seim

Norway

Olivier Seror

France

Ismail Sert

Turkey

Akiyoshi Seshimo

Japan

Delva Shamley

South Africa

Ron Shaoul

Israel

David Sherr

USA

Shuichiro Shiina

Japan

Sami Shimi

United Kingdom

Badri Man Shrestha

United Kingdom

Paolo Simioni

Italy

Primal Singh

New Zealand

Neil Smart

United Kingdom

Wade Smith

USA

Xiaoyan Song

USA

Cristiano Spadaccio

Belgium

Kai Sprengel

Switzerland

Philip Stahel

USA

Dorothee Staiger

Germany

Sigmar Stelzner

Germany

Oliver Strobel

Germany

Christine Stroh

Germany

Hasan Sunar

Turkey

Magnus Sundbom

Sweden

Randall Sung

USA

Nobumi Tagaya

Japan

Yuji Takakura

Japan

Kok-Yang Tan

Singapore

Konstantinos Tepetes

Greece

Oliver Michel Theusinger

Switzerland

Dietlind Tittelbach-Helmrich

Germany

Mauro Toppino

Italy

Christian Toso

Switzerland

Ervin Toth

Sweden

Christos Tourmousoglou

Greece

Jean Jacques Tuech

France

Altug Tuncel

Turkey

Laszlo Ungar

Hungary

Garth Utter

USA

Anil Vaidya

United Kingdom

Rahul Vaidya

USA

Cees Van Der Schans

Netherlands

Pieter Christiaan Van Der Sluis

Netherlands

Smruti Vartak

USA

Steven Velkes

Israel

Jean-Francois Vendrell

France

Joost Verhaagen

Netherlands

Akhil Verheyden

Germany

Gustavo Viani

Brazil

Andrew Vickers

USA

Ernst Von Dobschuetz

Germany

Urs Von Holzen

Switzerland

Jue Wang

USA

Zhifei Wang

USA

Collin Weber

USA

Theresia Weber

Germany

Ulrich Friedrich Wellner

Germany

Michael West

USA

Edward L. White

Israel

Corinna Wicke

Germany

Uwe Wittel

Germany

Evaghelos Xynos

Greece

Satoyoshi Yamashita

Japan

Hiroki Yamaue

Japan

Dao-Kuo Yao

China

Takushi Yasuda

Japan

Meryem Yilmaz

Turkey

Yingze Zhang

China

Fangjian Zhou

China

Martin Zielinski

USA

## Pre-publication history

The pre-publication history for this paper can be accessed here:

http://www.biomedcentral.com/1471-2482/14/2/prepub

